# Brieflets

**Published:** 1893-09

**Authors:** A. A. Burns

**Affiliations:** Smith's Falls, Ont.


ARTICLE V.

### BRIEFLETS.

BY A. A. BURNS, L. D. S., SMITH'S FALLS, ONT.

Where a cavity is found to contain deep-seated caries, and it has been found necessary to leave a portion remain, pressure upon the nerve may be prevented by applying a solution of chloro-percha to the remaining debris after it has been thoroughly treated with antiseptics. Allow the chloroform to evaporate, and a firm lining is found which is both non-conducting and non-irritating.

In filling an impression for a partial plate where there is danger of plaster teeth breaking, small wooden pegs, such as used by shoe-makers, placed in the tooth sockets with the plaster will prevent any such accident, and will also be found to give no resistance to cutting teeth down when about to imbed model in flask.

In finishing a cement filling after chemical action has taken place in setting process, a better finish may be obtained by rubbing the burnisher over a piece of white wax and then burnishing filling. This adds to the finish and prevents moisture from getting at filling for some time (although exposed to it), as a film of wax remains as a preventive.

A handy sand-paper mandrel may be made out of cork for use on rubber plates in finishing process. Take a common quart-taper cork. Trim up in cone shape on the lathe. By means of dental mechanical saw, make a slit through apical end of cone, about half way down. A piece of sand-paper, a little wider than the opening, bent back at one end for retention, and placed in the mandrel, will complete it. This will be found more pliable than common brass mandrel. It conforms more with depressions in outline of plate, retains sand-paper better and does not heat plate so quickly.

When a temporary root-filling is required, a good filling for such a case may be made by taking a small rope of absorbent cotton and saturating it in chloro-percha. This will be found very handy in removal, and will also prevent the continuance of decay of organic matter.

Good cotton holders and good disinfectant glass, for instruments, may be had by using small-size cut-flower jars. They can be obtained at any druggist's and will be found not only useful but ornamental. A great point in favor of these is that they are very easily kept clean.

In inserting a large amalgam crown-filling it is often found very hard to replace the cusps of the grinding surface. This may be accomplished by the use of the small round head tin tack, commonly called gimp tack. After the filling has been well retained, and cavity about half filled, a small hole may be made and one of these tin-tacks inserted at point where cusp is desired to be made. The amalgam can be packed around the tack, and it will be found to readily adhere to it on account of the affinity of mercury for tin. In a very large filling a couple of cusps may be had in a similar way. The great advantage in this way of cusp building is in the perfect articulation. The amalgam may be mixed a little soft, as the tin-tack will readily absorb the surplus mercury. After filling is in place, the patient is requested to close the teeth and the cusps will be found to conform perfectly with the accluding tooth. It is better to always have filling a little full, in this case, so that the pressure will give the above result. This makes one of the prettiest plastic fillings inserted, and experience has proven their durability.—*Dominion Dental Journal.*

